# Preparation and In Vitro Evaluation of Neutron-Activated, Theranostic Samarium-153-Labeled Microspheres for Transarterial Radioembolization of Hepatocellular Carcinoma and Liver Metastasis

**DOI:** 10.3390/pharmaceutics11110596

**Published:** 2019-11-12

**Authors:** Yin How Wong, Hun Yee Tan, Azahari Kasbollah, Basri Johan Jeet Abdullah, Chai Hong Yeong

**Affiliations:** 1 School of Medicine, Faculty of Health and Medical Sciences, Taylor’s University, Subang Jaya 47500, Selangor, Malaysia; yinhow.wong@taylors.edu.my (Y.H.W.); hunyee198@yahoo.com (H.Y.T.); bjja2016@gmail.com (B.J.J.A.); 2 School of Biosciences, Faculty of Health and Medical Sciences, Taylor’s University, Subang Jaya 47500, Selangor, Malaysia; 3 Medical Technology Division, Malaysian Nuclear Agency, Bangi 43000, Selangor, Malaysia; azahari@nuclearmalaysia.gov.my

**Keywords:** radioembolization, Samarium-153, microspheres, liver tumour, neutron activation, radiopharmaceutical

## Abstract

Introduction: Transarterial radioembolization (TARE) has been proven as an effective treatment for unresectable liver tumor. In this study, neutron activated, ^153^Sm-labeled microspheres were developed as an alternative to ^90^Y-labeled microspheres for hepatic radioembolization. ^153^Sm has a theranostic advantage as it emits both therapeutic beta and diagnostic gamma radiations simultaneously, in comparison to the pure beta emitter, ^90^Y. Methods: Negatively charged acrylic microspheres were labeled with ^152^Sm ions through electrostatic interactions. In another formulation, the Sm-labeled microsphere was treated with sodium carbonate solution to form the insoluble ^152^Sm carbonate (^152^SmC) salt within the porous structures of the microspheres. Both formulations were neutron-activated in a research reactor. Physicochemical characterization, gamma spectrometry, and radiolabel stability tests were carried out to study the performance and stability of the microspheres. Results: The Sm- and SmC-labeled microspheres remained spherical and smooth, with a mean size of 35 µm before and after neutron activation. Fourier transform infrared (FTIR) spectroscopy indicated that the functional groups of the microspheres remained unaffected after neutron activation. The ^153^Sm- and ^153^SmC-labeled microspheres achieved activity of 2.53 ± 0.08 and 2.40 ± 0.13 GBq·g^−1^, respectively, immediate after 6 h neutron activation in the neutron flux of 2.0 × 10^12^ n·cm^−2^·s^−1^. Energy-dispersive X-ray (EDX) and gamma spectrometry showed that no elemental and radioactive impurities were present in the microspheres after neutron activation. The retention efficiency of ^153^Sm in the ^153^SmC-labeled microspheres was excellent (~99% in distilled water and saline; ~97% in human blood plasma), which was higher than the ^153^Sm-labeled microspheres (~95% and ~85%, respectively). Conclusion: ^153^SmC-labeled microspheres have demonstrated excellent properties for potential application as theranostic agents for hepatic radioembolization.

## 1. Introduction

Liver cancer is the third leading cause of all cancer-related deaths globally and is responsible for approximately 8.2% of all cancers. Primary liver cancer, hepatocellular carcinoma (HCC), is the fifth most common cancer in men and eighth most common in women worldwide [1,2,3,4]. The mortality rate of primary liver cancer is high; only 5% of patients survive at 5 years after diagnosis, largely due to delayed diagnosis [5]. The liver is also a common metastatic site for other cancers, including colorectal, pancreatic, and stomach cancers. Colorectal cancer is especially prone to metastasize solely to the liver [6]. Approximately 50% of patients with colorectal cancer will develop liver metastasis during the course of their disease [7,8]. Surgical resection is the proposed curative treatment for good surgical candidates of liver cancer. Unfortunately, the symptoms of liver cancer often do not appear until it progresses to intermediate or advanced stages. In fact, more than three-quarters of patients are diagnosed during intermediate or advanced stages [2,9], where curative treatment is no longer an option for many patients. In reality, only 15% of patients are eligible for surgical procedures, while the rest of the patients undergo non-surgical therapies (50%) and best supportive care (35%) [5].

Transarterial radioembolization (TARE) is a minimally invasive procedure involving intra-arterial administration of radioembolic microspheres for the treatment of primary and metastatic malignancies in the liver. The procedure takes advantage of the dual blood supply to the liver (i.e., via the portal vein and hepatic artery). The hepatic artery primarily supplies blood to the neo-angiogenic tissues, while the portal vein supplies blood to approximately 75% of normal liver tissues [10]. In TARE, the radioembolic microspheres within the range of 20 to 60 µm are lodged in the capillary bed of liver tumors to deliver localized radiation doses, while sparing the surrounding healthy tissues [10]. With the expanding applications and continued interest in the use of radioembolic microspheres, the commercial development of Yittrium-90 (^90^Y) microspheres amenable for the treatment of liver cancer has been stimulated.

Currently, the glass-based TheraSphere^®^ (BTG, Ontario, Canada) and resin-based SIR-Spheres^®^ (SIRTex, North Sydney, Australia) microspheres are the common radioembolic agents for hepatic radioembolization. These radioembolic agents act as permanent brachytherapy implants and deliver the therapeutic dose (beta radiation) in situ after intra-arterial administration to the targeted tumor. Both TheraSphere^®^ and SIR-spheres^®^ utilize the same radionuclide, ^90^Y, as the source of therapeutic radiation. ^90^Y microspheres with favorable toxicity profiles have been established and proven by several tens of prospective or retrospective clinical studies to be safe and effective in treating primary and secondary liver cancers [11,12,13,14].

The ^90^Y radionuclide is commonly produced in a strontium-90 (^90^Sr) generator and is rarely produced through neutron activation from ^89^Y, because of its very low thermal neutron activation cross-section of ^89^Y. Separation of ^90^Y from ^90^Sr involves multiple steps, such as solvent extraction, ion exchange or/and extraction chromatography, and it produces a large amount of radioactive waste. Currently, ^90^Y-labeled microspheres are produced by industrial manufacturers. The cost is relatively high and the availability is limited because of transportation logistics. In addition, ^90^Y is a pure beta emitter, making the post-procedural imaging to verify the biodistribution of ^90^Y microspheres a challenge. Although bremsstrahlung imaging and positron emission tomography (PET) are often used for post-procedural imaging, both techniques face different challenges. For example, ^90^Y bremsstrahlung imaging produces low spatial resolution images caused by overlaying tissue attenuation, internal photon scattering, variable count rates of emitted bremsstrahlung photons, and the wide range of photon energies emitted. On the other hand, ^90^Y PET imaging requires relatively longer acquisition time because of its low true-coincidence count-rate for positron emission [15]. In addition, the high energy photons from bremsstrahlung X-rays may degrade the image quality substantially and limit the quantitative accuracy of ^90^Y PET imaging [15,16]. In order to improve detection efficiency, addition of other PET radiotracers, such as ^86^Y, has been suggested in previous studies [17,18,19]. However, similar to ^90^Y PET imaging, correction for spurious coincidences is mandatory in order to reduce overestimation errors resulting from random coincidences [17].

Verification of the microsphere distribution is crucial to prevent any procedural complications, such as hepatic dysfunction, biliary sequelae, radiation pneumonitis, gastroenteritis, and acute pancreatitis [20], and to estimate treatment outcome. A lung shunting study is usually performed before radioembolization using macro-aggregated albumin (MAA) labeled with Technetium-99m (^99m^Tc) to acquire desired information on the microsphere distribution within the liver and lungs. However, because of variations in physical characteristics and number of microspheres delivered during the lung shunting study and radioembolization treatment, the lung shunting study often fails to represent the intrahepatic distribution of ^90^Y microspheres accurately [21,22].

In view of the above limitations from ^90^Y microspheres, a theranostic radionuclide that emits both therapeutic beta and diagnostic gamma energies would be a suitable substitute for ^90^Y. A radionuclide with the following properties is regarded as an ideal theranostic radionuclide: an optimum physical half-life (several days to a week); suitable linear energy transfer (LET) and range in tissues; high ratio of non-penetrating to penetrating radiation; a short-lived or stable daughter; good and selective concentration in tumors, with prolonged retention; and minimum uptake by normal tissue [23]. In order to reduce the production cost, neutron-activated radionuclide is preferred because of its relatively simpler process, wider availability of nuclear reactors worldwide, and hence reduced cost for radioactive shipping.

Samarium-153 (^153^Sm) is a potential theranostic radionuclide for hepatic radioembolization. It has a physical half-life of 46.3 h; thermal neutron activation cross-section of 210 barns; and emits beta particles of 0.81 MeV (20%), 0.71 MeV (30%), and 0.64 MeV (50%), and gamma photons of 103 keV (28%) [24]. ^153^Sm can be produced via neutron activation by bombarding the stable parent nuclide, ^152^Sm, with a thermal neutron flux in a nuclear reactor. The reaction can be written as ^152^Sm(n,Υ)^153^Sm. Production of ^153^Sm with sufficiently high therapeutic activity and radionuclide purity using neutron activation has been studied extensively in the past [25,26]. ^153^Sm has been used for palliative pain treatment for bone metastases in the form of samarium lexidronam and radiosynovectomy of the knees. The application of ^153^Sm for gamma imaging has been demonstrated in some gastrointestinal transit and pharmacoscintigraphy studies [27,28,29]. However, its therapeutic potential for cancer treatment has not been fully utilized. Hashikin et al. investigated the physicochemical characteristics and radiation dosimetry of ^153^Sm-labeled microspheres for potential use in hepatic radioembolization [30,31,32]. Their study showed that ^153^Sm has the capability to deliver a tumor dose comparable to that of ^90^Y, while reducing the absorbed dose to the surrounding healthy liver tissues. Although ^153^Sm also emits gamma radiation that may penetrate and irradiate surrounding organs, the absorbed doses to all organs were well below 1 Gy [32]. However, Hashikin et al. failed to produce homogeneous spherical microspheres with a diameter in the range of 20–60 µm. Since the size of the radioembolic microspheres is an important feature of hepatic radioembolization, failure to produce the microspheres within the desired size range would compromise the safety of the treatment; the larger microspheres would not reach the capillary bed, while the smaller microspheres may pass through the tumor capillaries and reach the neighboring organs, especially the lungs [33].

Therefore, this study aimed to develop a ^153^Sm-labeled microsphere formulation with desired characteristics for hepatic radioembolization. In particular, the ^153^Sm-labeled microspheres should be within the size range of 20–60 µm, have a smooth and homogenous surface, can be activated via thermal neutron bombardment, should not produce radioactive impurities, should decay into stable nuclides, and should have strong chemical binding between ^153^Sm ions and the microspheres to prevent leaking of radioactive ^153^Sm from the microspheres [30]. In order to compare the ionic binding, two formulations were prepared in this study, namely ^153^Sm-labeled microspheres and ^153^SmC-labeled microspheres.

## 2. Materials and Methods

### 2.1. Preparation of Samarium-152 Labeled Microspheres

**Figure 1:**
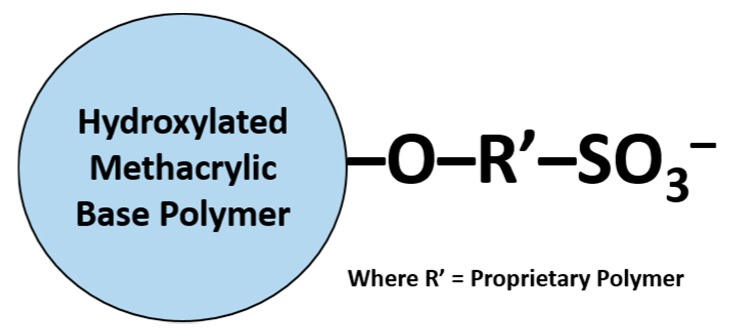
Chemical structure of the acrylic-based sulfopropyl microspheres.

Samarium-152 (III) chloride hexahydrate (^152^SmCl_3_.6H_2_O) with a natural isotopic abundance of 26.7% was purchased from Sigma-Aldrich Co., St. Louis, MO, USA. Commercially available acrylic-based microspheres attached to sulfopropyl groups (Toyopearl Gigacap 650s) were procured from Tosoh Corporation, Tokyo, Japan. The physicochemical properties and chemical structure of the microspheres are shown in Table 1 and Figure 1, respectively. The microspheres were washed five times with deionized water to eliminate the broken microspheres, sodium chloride, and ethanol. The microspheres were then dried in an oven at 70 °C for 12 h and stored in a desiccator.

Firstly, 1 g of ^152^SmCl_3_.6H_2_O was dissolved in 10 mL distilled water and 3 g of microspheres were added into the ^152^SmCl_3_ solution. The formulation was mixed homogenously using a magnetic stirrer and incubated for 15 min to allow binding of ^152^Sm^3+^ ions to the sulfopropyl groups of the microspheres. Then, the ^152^Sm-labeled microspheres were washed three times with distilled water and filtered using a 15 µm filter paper on the Büchner funnel filtration system to remove the unbound ^152^Sm^3+^ ions. The filtered ^152^Sm-labeled microspheres were dried in an oven at 70 °C for 12 h. The chemical equations for the preparation of the ^152^Sm-labeled microspheres are listed as follows:

HW65–O–R–SO_3_^−^*_(s)_* + ^152^Sm^3+^*_(aq)_* + 3 Cl^−^*_(aq)_* → (HW65–O–R–SO_3_)_3_^152^Sm *_(s)_* + 3HCl *_(aq)_* (1)

### 2.2. Preparation of Samarium-152 Carbonate Labeled Microspheres

The samarium-152 carbonate (^152^SmC)-labeled microspheres were prepared using the same methods mentioned above, except that the microspheres were treated with 3.5% (*w*/*v*) sodium carbonate (Na_2_CO_3_) solution after removing the unbound ^152^Sm^3+^ ions. The carbonate ions from the disassociation of Na_2_CO_3_ reacted with the ^152^Sm^3+^ ions and formed insoluble ^152^SmC within the porous structure of the microspheres. The ^152^SmC-labeled microspheres were then washed three times with distilled water and dried in an oven at 70 °C for 12 h.

### 2.3. Neutron Activation of ^152^Sm- and ^152^SmC-Labeled Microspheres

Neutron activation of the ^152^Sm- and ^152^SmC-labeled microspheres was done using a research reactor (TRIGA PUSPATI Reactor, TRIGA Mark II, General Atomics, San Diego, CA, USA) located at the Malaysian Nuclear Agency. The ^152^Sm- and ^152^SmC-labeled microspheres were activated using both the pneumatic transfer system (PTS) for 5 min and the rotary specimen rack (RR) method for 6 h to achieve a desirable radioactivity of 3 GBq [30]. The protocols for both neutron activation methods are given in Table 2. The irradiation time was calculated using Equation (2):

A_t_ = σ_act_ ϕ N (1 – e^−λt^) (2)

where; *A*_t_ = Activity at time t (Bq), σ_act_ = Thermal neutron activation cross-section (barns = 10^−24^ cm^2^), *N* = Number of parent atoms = (*m*/*w*) ×*θ* × 6.023 × 10^23^, *m* = Mass of the element in sample, *w* = Atomic weight of element, *θ* = Isotopic abundance, *λ* = Decay constant (s^−1^), *t* = Irradiation time (s).

The samples were kept for 48 h after neutron activation to allow for complete decay of the short-lived contaminate radionuclides. The activity of the activated samples was determined using a calibrated dose calibrator (CRC25R, Capintec Inc., Florham Park, NJ, USA). The specific activity was calculated by dividing the sample activity with the weight (in grams) of the microspheres.

### 2.4. Gamma Spectrometry

Gamma spectrometry was performed at 24 h and 48 h after neutron activation using a coaxial, p-type hyper-pure germanium (HpGe) detector (Canberra Inc., Meriden, CT, USA). Prior to the samples analysis, energy efficiency calibration was carried out to determine the detection efficiency of the HpGe detector at various gamma energies. Six standard gamma point sources (i.e., americium-241, cobalt-57, barium-133, europium-152, ceasium-137, and cobalt-60) with known energies and activities were used for the calibration. Each standard source was measured under identical geometry at three source detector distances of 5, 10, and 15 cm for a counting time of 300 s to obtain a dead time of less than 5%. The gamma spectra were then analyzed, and net areas under the gamma peaks (total count) for each of the considered gamma energies were determined. The energy efficiency, ε, was calculated according to Equation (3).

(3) $$\varepsilon =\;\frac{N}{\mathrm{Live}\;\mathrm{Time}\;\times \mathrm{Activity}\;\times \;\mathrm{Yield}}$$

where; *N* = net peak area (counts), Live time = the actual counting time (s), *A* = activity of the standard source (Bq), Yield is obtained from the certificate of the standard source.

The efficiency calibration curve (efficiency versus energy) was fitted to a polynomial function by the gamma spectrum analysis software (Genie^TM^ 2000 version 3.2, Canberra, Meriden, CT, USA). By referring to the calibration curve, the efficiency of the energy peaks detected from each sample was obtained and the activity of the sample could be estimated. Using this method, the presence of any radionuclide impurities in the sample could be identified.

### 2.5. Physicochemical Characterization of Sm- and SmC-Labeled Microspheres

#### 2.5.1. Field Emission Scanning Electron Microscopy and Energy Dispersive X-ray

Structural observation and validation of the chemical compositions of the Sm- and SmC-labeled microspheres before and after neutron activation were performed using a field emission scanning electron microscopy (FESEM) system (Quanta FEG 450, FEI, Hillsboro, OR, USA). The SEM images of the samples were obtained at 5 kV, 10 mm working distance, and spot size of 2.0. Validation of chemical compositions of the Sm- and SmC-labeled microspheres both before and after neutron activation were made using energy dispersive X-ray (EDX) spectroscopy on the FESEM system. The samples of microspheres were mounted on aluminum stubs before performing the SEM and EDX.

#### 2.5.2. Particle Size Analyzer

The mean particle size and particle size distribution were measured by a laser scattering particle size distribution analyzer (Microtrac X100, Microtrac Inc., Montgomeryville, PA, USA). Aliquots of Sm- and SmC-labeled microspheres were dispersed in distilled water by ultra-sonication, which was then loaded into the particle size analyzer.

#### 2.5.3. Fourier Transform Infrared (FTIR) Spectroscopy

Fourier transform infrared (FTIR) analysis of the Sm- and SmC-labeled microspheres was performed using a FTIR spectrometer (Spectrum 100, PerkinElmer Inc., Waltham, MA, USA) to investigate the effect of neutron activation on the different functional groups of the microspheres. The range was 600–4000 cm^−1^, with analysis performed both before and after neutron activation.

#### 2.5.4. Density Measurements

The particle density, ρ_s_, of Sm- and SmC-labeled microspheres was measured using a helium gas pycnometer (AccuPvc II 1340, Micromeritics Ins. Corp., Norcross, GA, USA) at standard room temperature of 25 °C. The ρ_s_ value was then incorporated into Equation (4) to estimate the number of microspheres.

(4) $$\mathrm{Number}\;\mathrm{of}\;\mathrm{microspheres}\;\mathrm{per}\;\mathrm{gram}=\;\frac{6\;\times \;10^{12}}{\pi \;\times {\;\rho }_{s}\;\times \;D_{p}{}^{3}\;}$$

where; *D_p_* = mean diameter of the microspheres (µm), ρ*_s_* = microspheres density (g·cm^−3^).

#### 2.5.5. Viscosity Measurements

The viscosity, *η*_o_, of 2.5% (*w*/*v)* microspheres in saline solution was measured at 37 °C, a using HAAKE™ MARS™ Rheometer (Thermo Fisher Scientific Inc., Waltham, MA, USA). The value was then incorporated into Stokes’ law (Equation (5)) to study the sedimentation rate (settling velocity) of the microspheres.

(5) $$V_{\mathrm{sed}\;=\;}\frac{g\;D_{p}{}^{2}\;\left(\rho_{s}\;-\;\rho_{f}\right)}{18\;\eta_{0}}$$

where; *V*_sed_ = sedimentation rate (cm·s^−1^), *g* = gravitational acceleration constant (981 cm·s^−2^), *η*_o_ = dynamic viscosity of the fluid (*P* = g·cm^−1^·s^−1^), ρ*_f_* = density of the fluid.

### 2.6. In Vitro Stability Test of the ^153^Sm- and ^153^SmC-Labeled Microspheres

The activated microspheres were transferred into 3 different glass tubes containers filled with 10 mL distilled water at a concentration of 2.5% (*w*/*v*). The tubes were then placed on a roller mixer (Movil-Rod, J.P. Selecta, Barcelona, Spain) and rolled at 50 rpm for 60 min. The samples were then centrifuged at 2000 rpm for 10 min. For each sample, 1 mL of the supernatant was transferred into a separate gamma assay tube. The procedures were repeated until a total of 8 mL of supernatant was obtained at sampling times of 1, 4, 24, 28, 48, 72, 96 and 120 h. The activity of the supernatant was assayed using a gamma scintillation counter (2470 Wizard2, PerkinElmer Inc., Waltham, MA, USA). The experiment was repeated in saline and human blood plasma. The human blood plasma was obtained from the Department of Transfusion Medicine, University Malaya Medical Centre (UMMC), Malaysia. Medical ethics approval was not required with reference to Scope 2.1.3, Standard Operating Procedure (SOP) of UMMC Medical Ethics Committee, since no donor personal identity information was acquired for this research. Hence, donor consent was also not required because of the anonymity of the sample. The retention efficiency of each formulation was calculated using Equation (6):

(6) $$\mathrm{Retention}\;\mathrm{Efficiency}\;\left(\%\right)\;=\;\frac{A_{\mathrm{sus}}\;-\;A_{\sup}}{A_{\mathrm{sus}}}\;\times 100$$

where; *A*_sus_ = Activity of microspheres suspension before each extraction of 1 mL supernatant, *A*_sup_ = Activity of 1 mL supernatant.

### 2.7. Statistical Analysis

The Mann-Whitley U test was used to compare the mean size of Sm- and SmC-labeled microspheres, and the differences were considered as significant when *p* < 0.05. All statistical analyses were performed using Statistical Product and Service Solutions (SPSS version 22.0, IBM, Armonk, NY, USA).

## 3. Results

### 3.1. Determination of Optimum Neutron Activation Protocol for ^152^Sm- and ^152^Smc-Labeled Microspheres

The ^152^Sm- and ^152^SmC-labeled microspheres were activated using both PTS and RR methods to determine the optimum neutron activation protocol, to achieve a desirable therapeutic radioactivity of 3.0 GBq for hepatic radioembolization. Although the sample delivery of PTS was more convenient and had three times higher neutron flux than RR (Table 2), it was limited by a maximum of 5 min irradiation time per sample. As a result of this limitation, the specific activities achieved using the PTS method for ^153^Sm- and ^153^SmC-labeled microspheres were 0.097 ± 0.006 GBq·g^−1^ and 0.086 ± 0.009 GBq·g^−1^, respectively, which were far below the target radioactivity of 3.0 GBq·g^−1^. The RR method using lower neutron flux but a longer irradiation time of 6 h produced specify activity values of 2.53 ± 0.08 GBq·g^−1^ and 2.40 ± 0.13 GBq·g^−1^ for ^153^Sm- and ^153^SmC-labeled microspheres, respectively. The physicochemical characteristics of the ^153^Sm- and ^153^SmC-labeled microspheres are given in Table 3. The activity values per microsphere for ^153^Sm- and ^153^SmC-labeled microspheres were 82.47 ± 2.60 and 77.77 ± 4.2 Bq, respectively (Table 3). Hence, the RR method was more suitable for the production of therapeutic ^153^Sm in this study.

### 3.2. Gamma Spectrometry of 153Sm- and 153Smc-Labeled Microspheres

**Figure 2:**
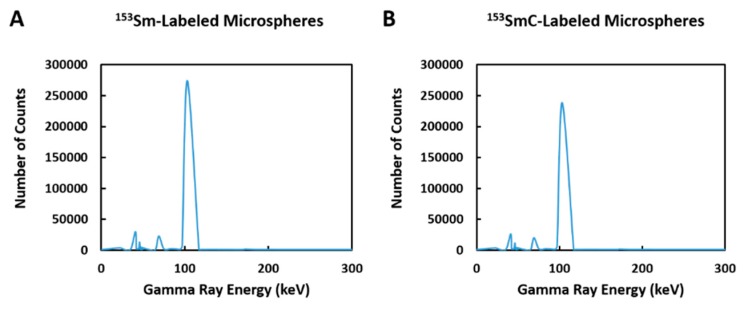
Gamma spectrum of ^153^Sm-labeled microspheres (**A**) and ^153^SmC-labeled microspheres (**B**) at 24 h post neutron activation.

Figure 2 shows the gamma spectrum of the ^153^Sm- and ^153^SmC-labeled microspheres. Four photopeaks (103.1 ± 0.2, 69.4 ± 0.2, 40.7 ± 0.2, and 46.5 ± 0.2 keV) were shown on the gamma spectra of both formulations. The two most dominant photopeaks (i.e., 103.1 and 69.4 keV) were the principal gamma energies emitted by ^153^Sm. On the other hand, the photopeaks at 40.7 and 46.5 keV resulted from the K-shell characteristic X-rays following internal conversion. Overall, no radionuclide impurity was observed in the ^153^Sm- and ^153^SmC-labeled microspheres at 24 and 48 h post neutron activation.

### 3.3. Effects of Neutron Activation on the Physicochemical Characteristics of ^152^Sm- and ^152^Smc-Labeled Microspheres

#### 3.3.1. Field Emission Scanning Electron Microscopy and Energy Dispersive X-ray

**Figure 3:**
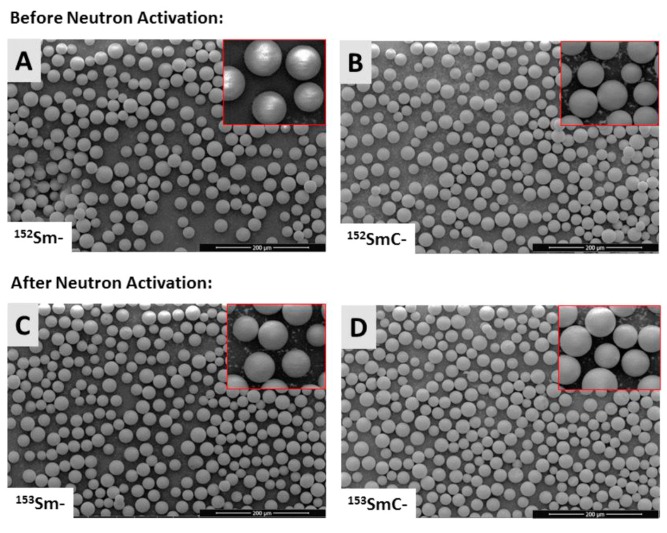
Scanning electron images of the Sm- and SmC-labeled microspheres both before (**A**,**B**) and after (**C**,**D**) 6 h neutron activation. The insets in the panels show the smooth surface morphology of the Sm- and SmC-labeled microspheres both before and after 6 h neutron activation, scale bar at the right bottom represent 200 μm.

Figure 3 shows the SEM images of the Sm- and SmC-labeled microspheres both before (Figure 3A,B) and after (Figure 3C,D) 6 h neutron activation. Both formulations remained spherical with smooth surface morphology before and after activation. In particular, formation of insoluble SmC salt within the porous structures of the microspheres did not alter the shape or surface morphology of the SmC-labeled microspheres (Figure 3B,D).

**Figure 4:**
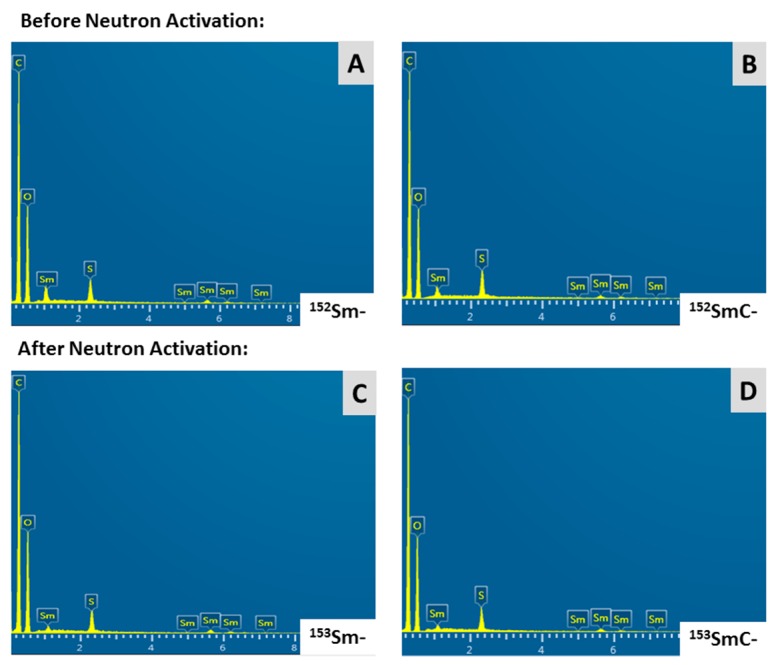
Energy dispersive X-ray (EDX) spectra of the of the Sm- and SmC-labeled microspheres both before (**A**,**B**) and after (**C**,**D**) 6 h neutron activation show the presence of chemical elements (i.e., carbon (C), oxygen (O), sulphur (S), and samarium (Sm)) in the samples. No chemical impurities were found after neutron activation.

The EDX spectra of the Sm- and SmC-labeled microspheres both before and after 6 h neutron activation are given in Figure 4. Since the microspheres used in this study are made of acrylic polymer with a sulfopropyl group, the chemical elements, such as carbon (C), oxygen (O), hydrogen (H), sulphur (S), and Sm, were expected to be present in both formulations. However, because of the limitation of EDX in detecting elements with atomic numbers lower than 6, the hydrogen was not shown in the EDX spectra. Figure 4 shows the presence of all the elements (C, O, S, and Sm) except for hydrogen, both before and after neutron activation. The elemental weight fractions of each sample suggested the presence of 7% Sm in Sm-labeled microspheres and 6% Sm in SmC-labeled microspheres. In other words, each 1 g of the Sm- and SmC-labeled microspheres consisted of 70 mg Sm and 60 mg Sm, respectively.

#### 3.3.2. Particle Size Analyzer

**Figure 5:**
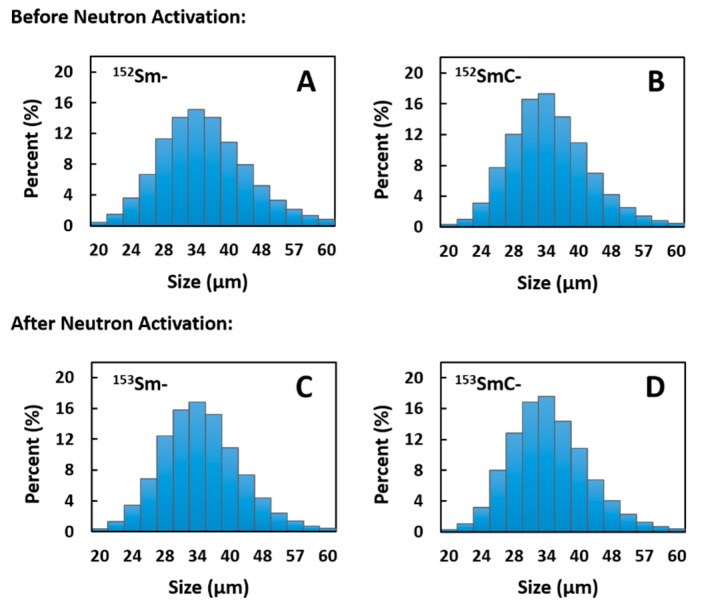
Particle size distribution of the Sm- and SmC-labeled microspheres both before (**A**,**B**) and after (**C**,**D**) 6 h neutron activation.

Figure 5 shows the particle size distribution of both formulations before and after neutron activation. The size distribution of the ^152^Sm- and ^152^SmC-labeled microspheres before neutron activation were within the range of 20–60 µm, with average diameters of 35.70 ± 0.15 µm and 35.63 ± 0.16 µm, respectively (Figure 5A,B, Table 3). Complexation of the SmC salt within the porous structure of the microspheres did not produce significant (*p* > 0.05) variation in the size distribution or average diameter of the microspheres. The neutron activation process did not significantly (*p* > 0.05) affect the size distribution of both formulations, where average diameters of the ^153^Sm- and ^153^SmC-labeled microspheres after neutron activation were 36.09 ± 0.15 µm and 35.62 ± 0.16 µm, respectively (Figure 5C,D, Table 3).

#### 3.3.3. FTIR Spectroscopy

**Figure 6:**
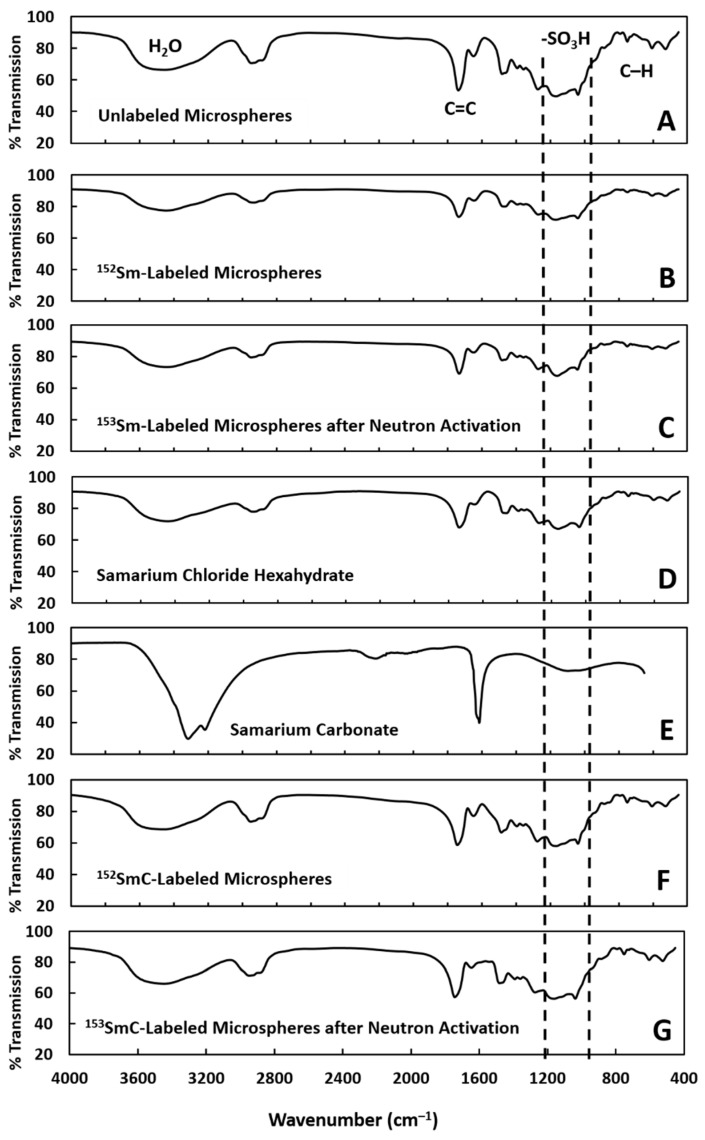
Fourier transform infrared (FTIR) spectra of the unlabeled microspheres (**A**), Sm-labeled microspheres (**B**) before and (**C**) after 6 h neutron activation, samarium chloride hexahydrate (**D**), samarium carbonate (**E**), and SmC-labeled microspheres (**F**) before and (**G**) after 6 h neutron activation.

The labeling of the ^152^Sm ions to the microspheres was solely dependent on the functionality and integrity of the negatively charged sulfopropyl groups. Hence, the integrity of the sulfopropyl groups of the microspheres after neutron activation would determine the retention of the ^153^Sm ions on the microspheres. FTIR spectroscopy was carried out to evaluate the effect of neutron activation on the structural integrity of the sulfopropyl groups to ensure that the retention efficiency did not decrease even after neutron activation. It can be seen from Figure 6 that the structure of the sulfopropyl groups remained intact after neutron activation. There were no major differences observed between those peaks before and after neutron activation. In other words, the neutron activation process did not produce any changes to the negatively charged groups, which was an important parameter to enable ^153^Sm ion binding.

#### 3.3.4. Density Measurement

The particle densities of the ^153^Sm- and ^153^SmC-labeled microspheres were found to be 1.3681 ± 0.0009 g·cm^−3^ and 1.3689 ± 0.0005 g·cm^−3^, respectively (Table 3).

#### 3.3.5. Viscosity Measurement

**Figure 7:**
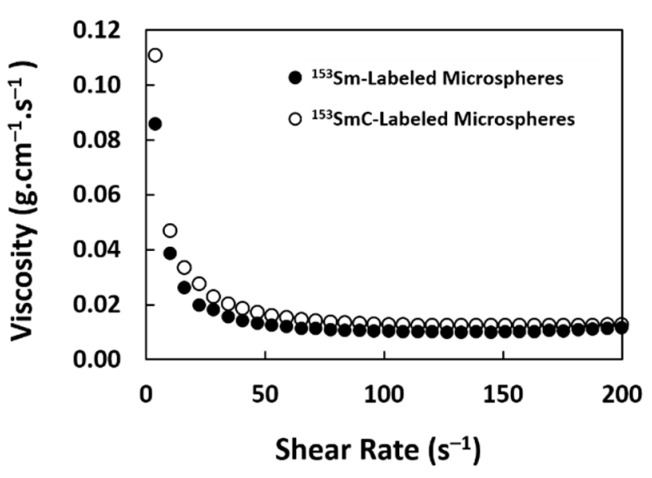
Viscosity of 2.5% *w*/*v* suspension of ^153^Sm- and ^153^SmC-labeled microspheres in saline at 37 °C with various shear rates.

The dynamic viscosities of the ^153^Sm- and ^153^SmC-labeled microspheres suspension at 37 °C are shown in Figure 7. Only a small difference in the viscosity was observed at various shear rates for both formulations; thus, the mean viscosity values of 0.0116 ± 0.00003 g·cm^−1^·s^−1^ and 0.0125 ± 0.00030 g·cm^−1^·s^−1^ were used to determine the settling velocity for the ^153^Sm- and ^153^SmC-labeled microsphere suspensions, respectively (Table 3). The estimated settling velocities were found to be 0.0154 ± 0.00005 cm·s^−1^ and 0.0168 ± 0.0004 cm·s^−1^ for the ^153^Sm- and ^153^SmC-labeled microspheres, respectively.

### 3.4. In Vitro Stability Test of the ^153^Sm- and ^153^Smc-Labeled Microspheres

**Figure 8:**
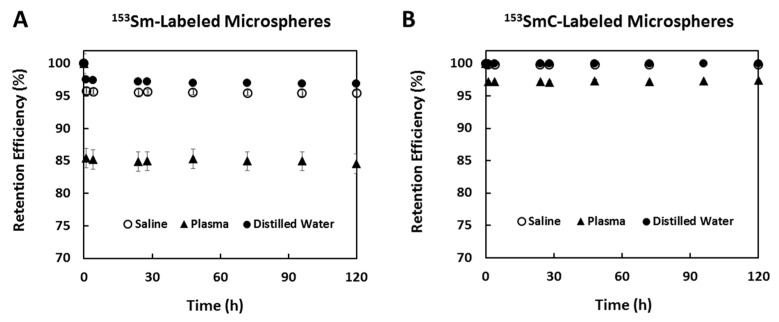
Retention (%) of ^153^Sm in ^153^Sm- (**A**) and ^153^SmC-labeled (**B**) microspheres suspended in distilled water, saline, and human blood plasma over 120 h.

The retention efficiency values of ^153^Sm in the ^153^Sm- and ^153^SmC-labeled microspheres in three different solutions, namely, distilled water, saline, and human blood plasma, are given in Figure 8. The retention of the ^153^Sm in the Sm-labeled microspheres decreased from 100% to ~97% in distilled water after 1 h incubation and plateaued for up to 120 h (Figure 8A). A similar observation was seen in saline and human blood plasma, but with lower retention of ~95% and 85%, respectively. The formation of insoluble ^153^SmC salt within the porous structures of the microspheres resulted in a much higher retention efficiency in the ^153^SmC-labeled microspheres (Figure 8B). The retention efficiency of the ^153^SmC-labeled microspheres in all three media were higher (~97–99%) than those observed in the ^153^Sm-labeled microspheres (~85–97%).

## 4. Discussion

Microspheres developed for hepatic radioembolization should be biocompatible, resistant to physical heat and chemicals found in the body, close to blood plasma density, and easily labeled with the targeted radionuclide. Ion exchange resins, which meet the above requirements, could feasibly be used as radioembolic microspheres [30]. The microspheres used in this study were cationic exchange resins with negatively charged groups that can bind with the positively charged ions through electrostatic interactions. The strength of the electrostatic interactions is generally stronger for ions with higher ionic charge and atomic number [34]. As such, the microspheres selected in this study were expected to have a higher affinity towards Sm^3+^ ions compared to other positively charged ions with lower ionic charge and atomic number.

Ion exchange resins have a long history as drug delivery carriers in various medical applications owing to their commercial availability, safety, and relatively easy preparation process [35,36,37]. In addition, because of their insoluble charateristic, the resins are not absorbed by the body, and thus, are safe for use in medicinal products with limited side effects [35,38]. Previous studies have demonstrated the potential use of acrylic- and styrene–divinylbenzene-based resins to radiolabel ^90^Y and holmium-166 (^166^Ho) [10,39,40] for hepatic radioembolization. The cationic exchange resin chosen in this study was made from a biocompatible acrylic polymer and is readily available with an average particle diameter of about 35 µm.

Neutron activation using the RR method has been determined as an optimum neutron activation protocol to produce ^153^Sm with high therapeutic activity close to 3 GBq·g^−1^. The nuclear reactor used in this study was a pool type TRIGA MARK II research reactor operated with 1 MW thermal power. The neutron flux used to irradiate the samples was 2.0 × 10^12^ n·cm^−2^·s^−1^. The activity prescribed for hepatic radioembolization can be manipulated by adjusting the neutron activation duration or the amount of the ^153^Sm- and ^153^SmC-labeled microspheres. It has been previously suggested [30] that administration of a larger number of microspheres with medium specific activity would achieve better therapeutic efficacy than a lower number of microspheres with higher specific activity. This is because a larger number of microspheres with medium specific activity is expected to distribute radiation doses more evenly within the tumor. Therefore, it is recommended to manipulate the number of microspheres rather than increase the neutron activation time in order to achieve higher total radioactivity for this study. In addition, the enriched form of ^152^SmCl_3_ (100% ^152^Sm) can be used to replace the natural abundance ^152^SmCl_3_ (~27% ^152^Sm) if higher specific activity is desired. The radioactivity produced by the enriched ^152^Sm is expected to be around three to four times higher than the naturally abundant ^152^Sm.

The physicochemical properties of the ^153^Sm- and ^153^SmC-labeled microspheres have not been altered by the neutron activation process. The maximum fuel temperature at the reactor core could reach up to 360 °C and the temperature at the water level is about 40 to 45 °C. The SEM results showed that both microspheres formulations remained spherical and smooth after 6 h neutron activation, indicating that the microspheres were able to sustain the high temperature environment in the nuclear reactor. The particle size distribution of both formulations also remained identical before and after neutron activation. The negatively charged sulfopropyl groups, which are important components in forming ionic bonds with the positively charged Sm ions, were also not altered by the neutron activation process. This was expected, as the chemical elements (e.g., ^32^S and ^16^O) in the sulfopropyl groups (–SO^3−^) of the microspheres require more than one neutron to be activated to radioactive isotopes, and hence they remained stable during the neutron activation process. This was further confirmed by the gamma spectrometry results.

The viscosity of the microsphere suspension was similar to the viscosity of the saline solution but was relatively lower than the viscosity of blood, which is about 0.03 g·cm^−1^·s^−1^ [41]. The diluted suspension at 2.5% was prepared to prevent blockage of the microcatheter during intraarterial delivery of the microspheres. The calculated settling velocity was relatively low, owing to the lower density of the microspheres compared to other polymeric microspheres. Since the settling velocity is mainly affected by the diameter and density of the material, the settling velocity of the microspheres developed in this study should perform better than the glass and resin-based microspheres commonly used in hepatic artery radioembolization [42,43,44]. The lower settling velocity would enable a more stable microsphere suspension, and hence the microspheres can be distributed more evenly within the tumor volume. This also prevents microspheres from settling in the microcatheter or blood vessels before reaching the tumor. The settling velocity can be further enhanced by using a solution with higher viscosity during the delivery, such as 5% dextrose solution. The usage of the 5% dextrose solution has been practiced in our institution for the administration of SIR spheres.

The retention of the radioactive ^153^Sm on the microspheres was studied in three solutions, namely, distilled water, saline, and human blood plasma. In clinical applications, the microspheres are suspended in distilled water during packaging to avoid ion displacement. The microspheres are then administered intra-arterially with the associated saline solution. The presence of other positive ions in the saline solution and blood plasma decreased the retention of ^153^Sm on ^153^Sm-labeled microspheres to ~95% and ~85%, respectively. The different ionic strengths of the saline solution and blood plasma might have weakened the ionic bonds between the ^153^Sm ions and negatively charged groups in the microspheres. In contrast, the ^153^SmC-labeled microspheres showed excellent retention of ^153^Sm in both saline and blood plasma (~97% to 99%). The formation of the insoluble ^153^SmC salt within the porous structure of the microspheres prevented the displacement of the ^153^Sm ions in saline and human blood plasma.

The insoluble carbonate salt was used in this study, as the carbon and oxygen elements in the carbonate salt had a smaller chance of being neutron activated in the nuclear reactor. As mentioned earlier, neutron activation of carbon and oxygen requires more than one neutron, and a previous study [30] has confirmed this finding. Although insoluble phosphate salt has been used in other studies [45,46], the phosphorus element in the phosphate salt has the potential to be neutron-activated into radioactive phosphorus-32 (^32^P), with a physical half-life of 14.29 days. Because of this concern, radioactive microspheres are usually produced first using either a generator or nuclear reactor prior to labeling of the insoluble phosphate salt. This added step increases radiation exposure to personnel and the cost of operation because of the requirement of additional shielding facilities. Thus, this study proposed the use of insoluble carbonate salt to enhance the labeling efficiency of ^153^Sm within the microspheres. Results from this study suggested that SmC-labeled microspheres had better labeling efficiency compared to Sm-labeled microspheres alone, and did not produce any radionuclide impurities.

In comparison to other studies, the main advantages of the microspheres produced in this study are the ability to produce zero radionuclide impurities with high ^153^Sm retention efficiency following a prolonged neutron activation process. Although the ^153^Sm-labeled microspheres showed a lower retention efficiency compared to Amberlite microspheres used in a previous study [30], this limitation was able to be solved by using insoluble SmC salt as a replacement. The ^153^Sm- and ^153^SmC-labeled microspheres tolerated the thermal stress in the nuclear reactor well and were not fragmented or damaged. The microspheres also preserved their great functionality despite long irradiation time, which was not possible for Poly (L-lactic acid) (PLLA) or other biodegradable polymers [47].

Production of radiolabeled microspheres using the neutron activation technique could possibly reduce the production cost and make the treatment more affordable across many nations. Current commercially available formulations for hepatic radioembolization are ^90^Y-labeled glass beads or resin microspheres. ^90^Y is produced by chemical high-purity separation from strontium-90 (^90^Sr), a fission product of uranium in nuclear reactors. The elution and labeling process involves multiple steps, using advanced technology and trained personnel. It also adds radiological burden to the staff as the labeling is done using radioactive ^90^Y, which emits high energy beta radiations. In comparison, the preparation of ^152^Sm- and ^152^SmC-labeled microspheres is relatively easier, cheaper, and does not involve ionizing radiation, as stable ^152^Sm is used during the labeling process. The complete formulation is then sent for neutron activation when needed. The irradiation time takes about 4 to 8 h depending on the reactor capacity and neutron flux. The formulation can be safely kept at the reactor site before dispatch. Currently, there are 224 operational research reactors in 53 countries, according to the latest International Atomic Energy Agency (IAEA) Research Reactor Database [48]. If the Sm- or SmC-labeled microspheres are locally produced, it would significantly reduce the transportation cost and lead time, benefiting more radioembolization patients. Some studies have suggested using neutron-activated rhenium-188 (^188^Re) and holmium-166 (^166^Ho) as substitutes to ^90^Y; however, these radioisotopes have relatively short half-lives of 17 h and 26 h, respectively. In our opinion, ^153^Sm has an ideal half-life of 46.3 h. Additionally, it emits both therapeutic beta energies (640, 710, and 810 keV) and diagnostic gamma radiation (103 keV, 29%), rendering it a useful theranostic agent for liver radioembolization. Furthermore, it is readily available in a stable parent form (^152^Sm, natural abundance 26%) and decays into a stable daughter (^153^Eu), minimizing the risk of radioactive waste.

## 5. Conclusions

In this study, ^152^Sm- and ^152^SmC-labeled microspheres with a mean size of 35 µm have been successfully synthesized using an ionic binding method in a standard chemistry lab. The formulations were non-radioactive until they were irradiated by thermal neutron flux in a research reactor. The physicochemical characteristics and morphology of the labeled microspheres were not altered by neutron activation. No significant radionuclide impurities were found in either formulation. ^153^SmC-labeled microspheres achieved higher labeling efficiency (97–99%) than ^153^Sm-labeled microspheres (85–97%). Therefore, the ^153^SmC-labeled microsphere was identified as a suitable formulation for liver radioembolization. Further studies are needed to verify in vivo pharmacokinetics and radiobiology properties using animal models before the formulation can be recommended for human use.

## Figures and Tables

**Table 1:** Product attributes of the Toyopearl Gigacap 650 s microspheres, as per the manufacturer’s specification.

Base Bead	Hydroxylated Methacrylic (HW)-65
Functional group	Sulfopropyl (S) strong cation exchange groups
Pore size (mean)	100 nm
Particle size (mean)	35 µm
Ligand type	strong cation
Ligand pKa	1.2
Dynamic binding capacity (DBC)	>150 g/L
Pressure rating	0.3 MPa
Shipping buffer	20% ethanol
pH stability	3–13
Shelf life (estimated)	10 years

**Table 2:** Neutron activation protocols using pneumatic transfer system (PTS) and rotary specimen rack (RR) methods.

Parameters	Pneumatic Transfer System (PTS)	Rotary Rack (RR)
Thermal Neutron Flux, θ_th_ (n·cm^−2^·s^−1^)	5.0 × 10^12^	2.0 × 10^12^
Irradiation Time	Maximum 5 min	Maximum 6 h
Irradiation Location	Near to the Core	Peripheral to the Core
Sample Delivery	Automatic	Manual

**Table 3:** Physicochemical characteristics of the **^153^**Sm- and **^153^**SmC-labeled microspheres.

Physicochemical Properties	153Sm-labeled Microspheres	153SmC-labeled Microspheres
Mean size (µm)	35.70 ± 0.15	35.63 ± 0.16
Density (g·cm^−3^)	1.3681 ± 0.0009	1.3689 ± 0.0005
Viscosity of 2.5% (*w*/*v*) microspheres suspension at 37 °C (g·cm^−1^·s^−1^)	0.0116 ± 0.00003	0.0125 ± 0.0003
Particle concentration (number of particles per g)	30,677,684	30,858,851
Specific activity (GBq·g^−1^)	2.53 ± 0.08	2.40 ± 0.13
Activity per microsphere (Bq)	82.47 ± 2.60	77.77 ± 4.2
